# Outcomes of a 42-day course of molnupiravir in cats with feline infectious peritonitis: a prospective descriptive study

**DOI:** 10.1093/jvimsj/aalag268

**Published:** 2026-10-28

**Authors:** Kenta Hishinuma, Akiko Inoue, Kenjiro Fukushima, Masahiko Sato

**Affiliations:** Veterinary Specialists Emergency Center, Saitama 333-0823, Japan; Veterinary Specialists Emergency Center, Saitama 333-0823, Japan; Veterinary Specialists Emergency Center, Saitama 333-0823, Japan; Veterinary Specialists Emergency Center, Saitama 333-0823, Japan

**Keywords:** antiviral therapy, feline infectious peritonitis, molnupiravir, nucleoside analogue, treatment duration

## Abstract

**Background:**

Antiviral therapy for cats with feline infectious peritonitis (FIP) has traditionally been administered for 84 days, although the optimal duration of molnupiravir treatment remains unknown.

**Hypothesis/Objectives:**

To evaluate the efficacy of a 42-day course of molnupiravir, orally (PO), for naturally occurring FIP in cats.

**Animals:**

Nineteen client-owned cats with newly diagnosed FIP.

**Methods:**

Prospective pilot study. Cats received molnupiravir, PO, q12h for 42 days at a median dosage of 11.5 mg/kg (IQR, 10.2-12.5 mg/kg). Cats with ocular or neurological involvement were excluded. Clinical assessments were performed during treatment and for 6 months after treatment cessation.

**Results:**

Nineteen cats were enrolled. Two cats failed to complete treatment because of death or persistent clinical signs. Fourteen of 19 cats (74%; 95% CI, 49-91) achieved sustained remission on an intention-to-treat basis. Each non-completer failed on day 5 or 10, respectively, within a window common to 42- and 84-day protocols, and therefore do not inform the question of treatment duration; among the 17 cats reaching day 42, 14 (82%; 95% CI, 57-96) remained in remission for at least 6 months. Three cats relapsed after treatment cessation; all responded to retreatment with molnupiravir at the same dosage extended to 84 days.

**Conclusions and clinical importance:**

This prospective study suggests that a shortened course of molnupiravir is feasible in selected cats with non-ocular, non-neurological FIP.

**Abstract (Japanese):**

背景: 猫伝染性腹膜炎 (FIP) に対する抗ウイルス療法は従来 84 日間実施されてきたが、モルヌピラビルの最適な治療期間は明らかではない。 目的: 自然発症 FIP の猫に対するモルヌピラビル 42 日間経口投与の有効性を評価すること。 動物: 新たに FIP と診断された飼育猫19頭。 方法: 前向きパイロット研究。モルヌピラビルを 12 時間ごとに 42 日間経口投与した。投与量の中央値は 11.5  mg/kg (四分位範囲、 10.2-12.5  mg/kg) であった。眼病変または神経学的病変を有する猫は除外した。治療中および治療終了後 6 か月間、臨床評価を行った。 結果: 19 頭を登録した。 2 頭は死亡または臨床徴候の持続により治療を完遂できなかった。 Intention-to-treat 解析では 19 頭中 14 頭(74 %； 95% 信頼区間、 49-91) が持続的寛解を達成した。治療非完遂の 2 頭はそれぞれ5日目および10日目に治療不成功となり、いずれも 42 日間および 84 日間の両治療プロトコルに共通する期間内であったため、治療期間の検討には寄与しなかった。 42 日目まで治療を継続した 17 頭のうち 14 頭(82% ； 95% 信頼区間、 57-96) は少なくとも 6 か月間寛解を維持した。 3 頭は治療終了後に再発したが、全頭が同一用量のモルヌピラビルによる 84 日間の再治療に反応した。 結論および臨床的重要性: 本前向きパイロット研究の結果から、眼病変および神経学的病変を伴わない FIP の猫では、モルヌピラビルの短期治療が有効である可能性が示唆された。.

## Introduction

Feline infectious peritonitis (FIP) was historically considered a uniformly fatal disease resulting from mutation of feline coronavirus (FCoV) into pathogenic strains capable of replicating within monocytes and macrophages, leading to dysregulated immune responses and severe systemic inflammation.^1^

In recent years, nucleoside analog antivirals have fundamentally changed the clinical outlook for cats with FIP. These agents inhibit viral replication, induce rapid clinical improvement and long-term survival, and have transformed FIP into a treatable and potentially curable disease.^2^ GS-441524 and its prodrug remdesivir (GS-5734) were the first to demonstrate high efficacy and favorable safety in experimental and clinical studies, establishing nucleoside analog therapy as the cornerstone of modern FIP treatment.^3^^,^^4^

Molnupiravir, another nucleoside analog originally developed for human viral infections, is an effective treatment for FIP.^5^ GS-441524 and molnupiravir as first-line treatments have comparable efficacy and safety, supporting molnupiravir as a viable alternative antiviral therapy.^6^ However, GS-441524 remains the preferred first-line treatment because molnupiravir might have a narrower therapeutic window, is associated with adverse effects such as neutropenia, and there are theoretical concerns regarding viral mutation and antiviral resistance.^7^

Traditionally, antiviral treatment for FIP is administered for 12 weeks based on early treatment protocols and subsequent clinical experience.^3^^,^^4^^,^^8^ However, the optimal treatment duration has not been established. Prolonged therapy increases financial burden, complicates medication administration, and could reduce caregiver compliance and quality of life for both cats and caregivers. Shorter treatment durations could also minimize unnecessary drug exposure and the potential risk of antiviral resistance. In human medicine, antiviral therapy for RNA viral infections is typically administered for only 3-5 days once clinical improvement is achieved.^9^ However, because FIP differs markedly from human respiratory coronavirus infections in its pathogenesis and clinical course, its optimal treatment duration remains uncertain.

Forty-two-day and 84-day courses of GS-441524, orally (PO), in cats with effusive FIP have comparable outcomes, suggesting that a shorter course might be sufficient.^10^ Shortened treatment protocols could therefore reduce treatment costs and the burden of prolonged daily medication.

Despite these findings, whether a shortened treatment duration is similarly effective for molnupiravir, particularly in cats with non-effusive FIP, remains unknown. Therefore, the objective of this study was to report the efficacy of a 42-day course of molnupiravir, PO, in cats with naturally occurring effusive and non-effusive FIP.

## Materials and methods

This was a prospective observational study conducted at a referral center between 2023 and 2025. Client-owned cats with clinically diagnosed FIP were enrolled after caregiver consent. The study protocol was approved by the ethical committee of our institution (approval number R71127-1).

Cats exhibited signalment, clinical signs, and disease progression consistent with FIP based on the Advisory Board on Cat Diseases guidelines.^11^ A presumptive diagnosis was established by an American College of Veterinary Internal Medicine board-certified internist using clinicopathologic testing, radiography, ultrasonography, cytology, FCoV reverse transcription polymerase chain reaction (RT-PCR) performed at IDEXX Laboratories, and overall clinical assessment. Cats with negative RT-PCR results were included if clinical findings were consistent with FIP and sustained clinical improvement was observed after antiviral therapy.

Cats with overt ocular lesions or signs of neurological disease were excluded because these forms are associated with poorer outcomes and often require longer treatment durations.^12^^,^^13^ Therefore, these cases were excluded from the short-course treatment protocol. Additional exclusion criteria included caregiver selection of alternative treatment protocols (GS-441524 or an 84-day molnupiravir regimen) and positive feline immunodeficiency virus or feline leukemia virus status.

All cats received molnupiravir monotherapy, PO, (Molnuvid 200; Healing Pharma India Pvt. Ltd.) at a dosage of 10-15 mg/kg, compounded by a pharmacist, every 12 h for a fixed duration of 42 days. The purity or composition of the compounded molnupiravir preparation was not independently verified. Supportive treatments, including fluid therapy and appetite stimulants, were permitted at the clinician’s discretion. Dosage adjustments were made based on changes in body weight at follow-up visits.

Re-evaluations were performed at weeks 1, 3, and 6 during treatment and at months 1, 2, 3, and 6 after treatment cessation. Each visit included a physical examination, complete blood count, serum biochemistry profile, and point-of-care in-house serum amyloid A measurement (SAA), as previously reported.^14^ Ultrasonography was performed during treatment and follow-up as clinically indicated.

The primary outcome was treatment success, defined as the absence of FIP-related clinical signs for at least 6 months after treatment cessation. Relapse was defined as recurrence of clinical signs consistent with FIP after treatment discontinuation.

Statistical analyses were performed using StatMate V (Atoms). Linear mixed-effects models were used to assess changes in hematologic variables over time. Statistical significance was set at *P* < .05.

## Results

### Enrollment

A total of 32 cats were diagnosed with FIP during the study period. Of these, 13 cats were excluded due to suspected ocular or neurological FIP (4 cats), caregiver selection of an alternative treatment protocol (8 cats), or loss to follow-up associated with poor owner compliance (1 cat). Nineteen cats that met the inclusion criteria were enrolled in the study. The signalment of the cats is summarized in Table 1. At enrollment, 10 cats were classified as having effusive FIP (7 cats with abdominal effusion, 2 cats with both pleural and abdominal effusions, and 1 cat with pleural effusion), while 9 cats were classified as having non-effusive FIP (4 cats with enlarged abdominal lymph nodes, 2 cats with multiple peritoneal nodules and thickening, 2 cats with enlarged abdominal lymph nodes and a colonic mass, and 1 cat with enlarged abdominal lymph nodes and renomegaly). Sixteen cats tested positive for FCoV by RT-PCR in effusion samples, enlarged lymph nodes, or mass lesions. In the remaining 3 cats, RT-PCR testing of lesions was negative; however, a diagnosis of FIP was established based on concordant clinical findings and a positive, sustained response to treatment. Clinical signs and clinicopathological abnormalities at enrollment are summarized in Tables 2 and 3.

**Table 1:** Summary of baseline characteristics of cats included in this study.

**Age at diagnosis, years, median (range)**	2.6 (0.5-11.9)
**Breed, *n* (%)**	
**Pure**	13 (68)
**Domestic shorthairs**	6 (32)
**Sex, *n* (%)**	
**Male intact/castrated, *n* (%)**	5 (26)/8 (42)
**Female intact/spayed, *n* (%)**	0 (0)/6 (32)
**Duration from disease onset to treatment initiation, days, median (range)**	15 (2-91)
**Type, *n* (%)**	
**Effusive**	10 (52)
**Non-effusive**	9 (48)

**Table 2:** Clinical signs observed at enrollment.

**Clinical signs**	**percentage**
**Loss of appetite**	73
**Lethargy**	68
**Fever**	68
**Distended abdomen**	26
**Tachypnea**	21
**Polyuria/Polydipsia**	12
**Nasal discharge**	12
**Diarrhea**	6

**Table 3:** Clinicopathological data at enrollment.

**Clinicopathological data**	**Median (IQR)**	**Reference interval**	**Abnormal percentage**
**Serum amyloid A (μg/mL)**	63.4 (30.0-165.9)	<5.5	89
**Lymphocytes (×10^3^/μL)**	0.5 (0.4-1.5)	1.5-7.0	79
**Total protein (g/dL)**	8.4 (7.1-9.3)	5.7-7.8	68
**Globulin (g/dL)**	5.8 (5.1-6.8)	2.7-5.2	68
**Neutrophils (×10^3^/μL)**	13.5 (8.1-17.0)	2.5-12.5	63
**Bilirubin (mg/dL)**	0.3 (0.2-1.2)	0.1-0.4	47
**Cholesterol (mg/dL)**	168 (145.5-256.5)	89-176	47
**Triglyceride (mg/dL)**	102 (68-184)	17-104	47
**Platelets (×10^3^/μL)**	265 (90.5-559)	200-800	47
**PCV (%)**	28 (22.5-33)	24-45	37
**ALP (U/L)**	35 (25-54)	0-58	21
**Albumin (g/dL)**	2.5 (2.1-2.7)	2.3-3.5	21
**ALT (U/L)**	52 (36-77)	22-84	16

### Treatment response and outcome

The median dose of molnupiravir was 11.5 mg/kg (interquartile range [IQR], 10.2-12.5 mg/kg). One cat with effusive FIP died suddenly 5 days after initiation of therapy while receiving molnupiravir at 10.1 mg/kg twice daily. Because a necropsy was not performed, the cause of death could not be determined. Another cat with non-effusive FIP received molnupiravir at a dose of 12.9 mg/kg twice daily; however, fever and lethargy persisted on day 5 and the molnupiravir dose was subsequently increased to 18 mg/kg twice daily, but no clinical improvement was observed. Therefore, the treatment protocol was changed to GS-441524 on day 10, after which the cat responded favorably to therapy.

Seventeen cats completed the 42-day course of molnupiravir and underwent scheduled follow-up monitoring for at least 6 months after treatment discontinuation.

Administration of molnupiravir resulted in consistent clinical and clinicopathological improvement in the 17 cats. Clinical signs improved by the first recheck visit. By week 2 of treatment, 8 of the 9 cats with effusion at diagnosis showed marked reduction in pleural or abdominal effusion, while abdominal effusion persisted in the remaining cat but demonstrated clear improvement by week 6. Ultrasonographic abnormalities—including lymphadenopathy, colonic mass, pleural or peritoneal thickening, renomegaly and intra-abdominal nodular lesions—resolved rapidly; the resolution was observed in 10 cats by week 3 and in the remaining 7 cats by week 6.

Hematologic and biochemical abnormalities improved after initiation of treatment (Figure 1). White blood cell counts, SAA, and bilirubin concentrations decreased significantly (*P* < .05) by the 1-week recheck, whereas an increase in packed cell volume (PCV) required up to 3 weeks. Serum globulin concentrations decreased significantly (*P* < .05) by the 6-week recheck; however, complete resolution of hyperglobulinemia required up to 3 months after treatment discontinuation in 1 case.

**Figure 1:**
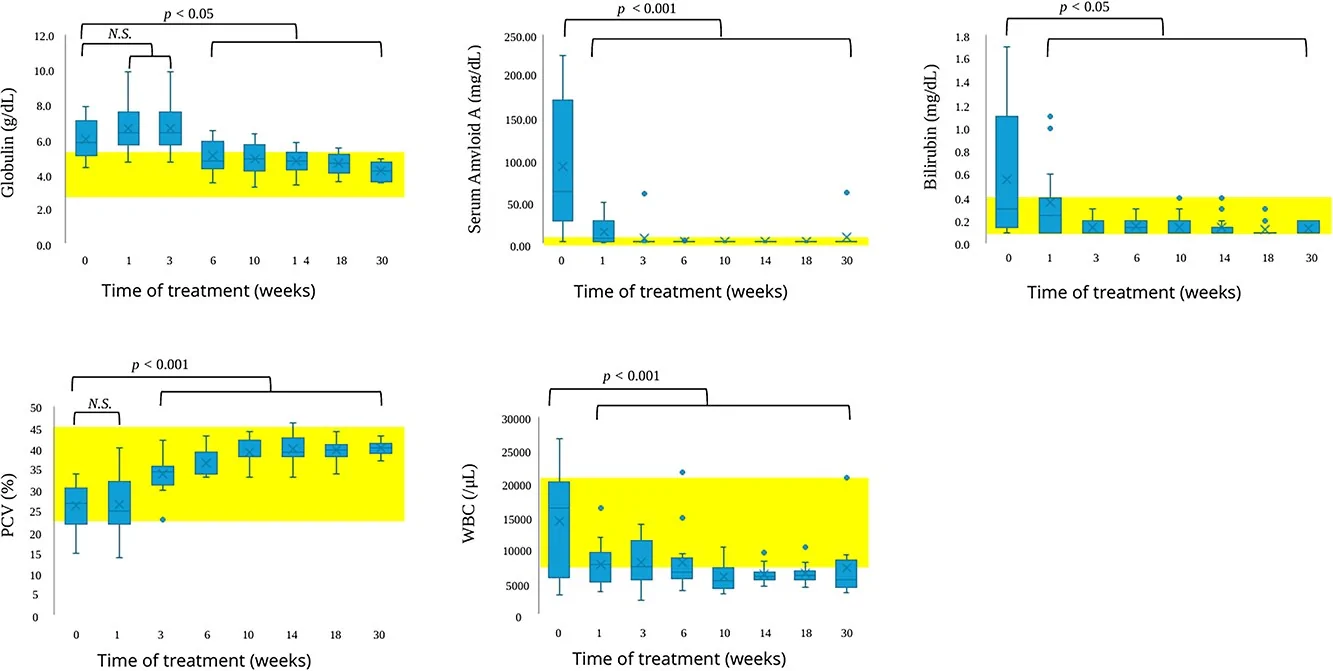
Clinicopathological variables during the follow-up period. The gray background indicates the reference intervals. Boxes represent the interquartile range, the horizontal line within each box indicates the median, and the cross marks indicate the mean values. Whiskers represent the minimum and maximum values, and outliers are shown as filled circles. P values shown in the figure were calculated using a linear mixed model. Abbreviations: NS = not statistically significant; PCV = packed cell volume; WBC = white blood cell count.

Fourteen cats maintained remission for at least 6 months after treatment discontinuation. The median follow-up duration after treatment cessation among cats that maintained remission was 26 weeks (range, 26-29 weeks). Three cats developed recurrence of clinical signs, including fever, lethargy, and anorexia, at 21, 27, and 91 days after cessation of treatment, respectively. One cat with effusive FIP developed recurrent pleural effusion, and RT-PCR for coronavirus was positive in the effusion sample. Another cat with non-effusive FIP showed enlargement of the abdominal lymph nodes, and RT-PCR for coronavirus was positive in the lymph node aspirate sample. The third cat with non-effusive FIP, which initially presented with abdominal lymphadenopathy and renomegaly, developed fever accompanied by increased SAA and globulin concentrations on bloodwork. However, abdominal ultrasonography did not reveal any obvious abnormalities at the time relapse was suspected, and RT-PCR testing was not performed in this case. The cat was initially treated with fluid therapy and an appetite stimulant for 5 days; however, the clinical signs did not improve. FIP relapse was still suspected, and the clinical signs resolved within 1 day after restarting molnupiravir therapy. Therefore, all 3 cats were classified as relapse cases. In 2 cats that relapsed at 21 and 27 days after treatment cessation, no interim recheck data were available between discontinuation and relapse. In the cat that relapsed at 91 days after treatment cessation, re-evaluation data were available at 1, 2, and 3 months after discontinuation. In this cat, serum globulin concentrations increased gradually after cessation of therapy, rising from 4.2 g/dL at the end of treatment to 5.0, 5.2, and 5.5 g/dL at the 1-, 2-, and 3-month rechecks, respectively, before the development of clinical signs 7 days after the final recheck. No other clinicopathological changes predictive of relapse were identified at 1, 2, or 3 months after treatment cessation.

All 3 relapse cases were restarted on molnupiravir at the same dosage as the initial treatment (10.2, 11.5 and 14.3 mg/kg q12h, respectively) and treatment duration was extended to approximately 84 days. All cats showed rapid clinical improvement, and antiviral therapy was discontinued after completion of the extended course. All cats maintained remission for more than 6 months after retreatment. The signalment, FIP type, molnupiravir dosage, and treatment outcomes are summarized in the Supplementary material.

### Adverse events

Mild, asymptomatic increases in alanine aminotransferase (ALT) activity, ranging from 100 to 200 U/L (reference intervals: 22-84 U/L), were observed in 5 of 19 exposed cats (26%) after initiation of molnupiravir therapy and were monitored without intervention. These elevations resolved either during treatment or after treatment cessation.

Neutropenia was observed in 2 of 19 cats (11%) after initiation of molnupiravir therapy, with neutrophil counts ranging from 700/μL to 1490/μL (reference intervals: 2500-12 500/μL). No cats developed clinical signs attributable to neutropenia, and antimicrobial therapy was not administered. Neutrophil counts returned to within the reference interval by the 1-month recheck after molnupiravir cessation.

No other adverse events, such as signs of gastrointestinal disease, hair loss, or ear folding, were observed.

## Discussion

This prospective study reports that a 42-day course of oral molnupiravir monotherapy was effective and well tolerated in cats with naturally occurring FIP. Among the 19 cats included in this study, 2 cats (11%) failed to achieve remission and did not complete the 42-day treatment protocol, 3 cats (16%) relapsed after completing the 42-day protocol, and 14 cats (74%) achieved long-term remission after the initial 42-day treatment course.

Eleven percent of cats treated with GS-441524 died without achieving remission after the initial treatment course,^15^ and that relapse rates after completion of an 84-day GS-441524 treatment regimen ranged from 0% to 16%.^3^^,^^4^^,^^6^^,^^16^ A recent prospective study evaluating an 84-day molnupiravir treatment protocol with or without an immune stimulant reported that 23% of cats failed to complete the 84-day treatment course,^17^ and relapse rates range from 0% to 19% after completion of an 84-day molnupiravir treatment regimen.^5^^,^^6^^,^^17^^,^^18^ The variability in reported relapse rates is likely attributable, at least in part, to institutional differences in case populations, such as private practices versus referral hospitals, where more severe or advanced cases are more commonly encountered. The timing of treatment initiation and access to treatment might also influence the outcome. Although direct comparisons across studies are not possible, the point estimates for long-term remission and relapse fell within the ranges reported previously. The relapse proportion of 3/17 (18%) carries a 95% CI of 4%-43%, an upper bound well above the 0%-19% range reported after 84-day regimens; these data are therefore compatible with either equivalent or substantially inferior performance and do not establish non-inferiority. This study supports previous findings suggesting that a 42-day course of GS-441524 demonstrates efficacy comparable to that of an 84-day course in cats with effusive FIP.^10^ Importantly, our study also included cats with non-effusive FIP and demonstrated that a shortened treatment course was effective in these cases as well.

Improvement in clinical signs was observed within one week of treatment initiation, consistent with previous reports.^11^ In contrast, normalization of PCV and serum globulin concentrations required a longer period than that of SAA, total bilirubin, and neutrophil counts after initiation of therapy; therefore, PCV and globulin concentrations might be less suitable as early markers of treatment response. SAA was evaluated in this study because in-house SAA measurement was available at our hospital. In the present cohort, SAA was more frequently elevated (89%) at the time of FIP diagnosis than other clinicopathological parameters, including total bilirubin concentration (47%), neutrophil count (63%), and globulin concentration (68%). In addition, SAA concentrations declined rapidly after treatment initiation, suggesting that SAA is a useful and objective biomarker for monitoring early clinical response to antiviral therapy, consistent with the findings in the previous study.^10^^,^^19^

Relapse occurred between 3 and 13 weeks after treatment cessation, consistent with previously reported relapse intervals.^3^^,^^4^ All relapsed cats responded favorably to retreatment with molnupiravir at the same dosage, and sustained remission was achieved after extension of the treatment duration. These findings suggest that relapse does not necessarily indicate the development of antiviral resistance, but might instead be associated with persistence of viral infection within tissues. Although dose escalation is often recommended for relapse, all 3 cats in the present study responded to retreatment with the same molnupiravir dosage combined with an extended treatment duration. However, the optimal retreatment strategy for cats that relapse after treatment cessation remains unknown.

Adverse events associated with molnupiravir were infrequent and generally mild. Transient, asymptomatic increases in ALT activity and neutropenia were observed but did not result in clinical complications or necessitate treatment discontinuation. These findings are consistent with reports of good tolerability of molnupiravir administered at similar or higher dosages and for longer treatment durations.^5^^,^^6^^,^^8^^,^^17^

The median age of cats in this study was older than that typically reported for cats with FIP, which are usually younger than 2 years of age.^11^ The reason for this difference is unclear, but it might reflect differences in the study sample and referral bias. As antiviral therapies have become more widely available in general practice, more typical and straightforward FIP cases can now be diagnosed and treated without referral, resulting in an older and potentially more complex referral sample in this study.

Cats with ocular or neurological FIP were excluded from this study because these forms are associated with less favorable outcomes and typically require longer treatment durations.^12^^,^^13^ Consequently, it remains unclear whether a short-course molnupiravir regimen would be equally effective in cats with ocular or neurological FIP.

This study has several limitations. The sample size was small. Ophthalmologic and neurological examinations were performed in all cases; however, mild ocular or neurological abnormalities associated with FIP might have been overlooked. The purity and composition of the compounded molnupiravir product were not independently verified. At the time this study was initiated, evidence supporting the efficacy of a shortened treatment course of GS-441524 was not yet available; therefore, this investigation was designed as a pilot study, with the intent of informing subsequent randomized trials should comparable outcomes be observed. The absence of a control group receiving an 84-day molnupiravir regimen represents an important limitation. Larger, controlled prospective randomized studies are warranted to further define the optimal treatment duration of molnupiravir for FIP.

## Conclusions

In this prospective study of cats with non-ocular, non-neurological FIP, a 42-day course of molnupiravir resulted in sustained remission in 14 of 19 cats (74%).

## Supplementary Material

FIP_Supplemental_data_aalag268

## Data Availability

The data supporting the findings of this study are available from the corresponding author upon reasonable request.

## Off-label antimicrobial declaration

Molnupiravir was used off-label for the treatment of feline infectious peritonitis.

## Institutional animal care and use committee or other approval declaration

The study protocol was approved by the Animal Ethics Committee of Veterinary Specialists Emergency Center (approval number R71127-1). Written informed consent was obtained from all owners prior to enrollment of their cats in the study.

## Abbreviations

CBC: complete blood count
FCoV: feline coronavirus
FIP: feline infectious peritonitis
PCR: polymerase chain reaction
SAA: serum amyloid A
