# Supplementary material for: Outcomes of a 42-day course of molnupiravir in cats with feline infectious peritonitis: a prospective descriptive study
Source: J Vet Intern Med. 2026 Oct 28;40(5):aalag268. doi: 10.1093/jvimsj/aalag268 (PMC13714784; doi:10.1093/jvimsj/aalag268)
Supplement: Supplementary file 1 [file fip_supplemental_data_aalag268.docx]

Supplemental Data: Signalment, FIP type, molnupiravir dosage, and treatment outcomes of cats with FIP included in this study.

| Breed | Age (year) | Sex | FIP type | Dosage  (mg/kg/bid) | Outcomes |
| --- | --- | --- | --- | --- | --- |
| American shorthair | 8 | MC | Non-effusive | 12.0 | Completed a 42-day MPV without relapse during the follow-up |
| Maine coon | 2.8 | FS | Effusive | 10.2 | Completed a 42-day MPV without relapse during the follow-up |
| Somali | 7.0 | MC | Effusive | 10.0 | Completed a 42-day MPV without relapse during the follow-up |
| Scottish fold | 1.0 | FS | Effusive | 11.0 | Completed a 42-day MPV without relapse during the follow-up |
| Scottish fold | 0.6 | M | Effusive | 10.0 | Completed a 42-day MPV without relapse during the follow-up |
| Russian blue | 0.6 | M | Effusive | 12.1 | Completed a 42-day MPV without relapse during the follow-up |
| Scottish fold | 3.3 | M | Effusive | 10.4 | Completed a 42-day MPV without relapse during the follow-up |
| Domestic shorthair | 11.9 | M | Non-effusive | 10.3 | Completed a 42-day MPV without relapse during the follow-up |
| Munchkin | 5.0 | MC | Non-effusive | 12.5 | Completed a 42-day MPV without relapse during the follow-up |
| Munchkin | 0.6 | FS | Non-effusive | 12.0 | Completed a 42-day MPV without relapse during the follow-up |
| Siberian | 0.5 | M | Non-effusive | 13.1 | Completed a 42-day MPV without relapse during the follow-up |
| Domestic shorthair | 2.0 | MC | Effusive | 15.3 | Completed a 42-day MPV without relapse during the follow-up |
| Siberian | 2.6 | FS | Effusive | 11.6 | Completed a 42-day MPV without relapse during the follow-up |
| Domestic shorthair | 5.3 | MC | Non-effusive | 11.2 | Completed a 42-day MPV without relapse during the follow-up |
| Minuet | 6.3 | MC | Non-effusive | 14.3 | Completed a 42-day MPV; relapsed 21 days post-treatment |
| Domestic shorthair | 1.6 | MC | Effusive | 11.5 | Completed a 42-day MPV; relapsed 27 days post-treatment |
| Domestic shorthair | 1.6 | FS | Non-effusive | 10.2 | Completed a 42-day MPV; relapsed 91 days post-treatment |
| American shorthair | 0.6 | MC | Non-effusive | 12.9 | No response to MPV and switched to GS on day 10 |
| Domestic shorthair | 11.3 | FS | Effusive | 10.1 | Sudden death on day5 |

M: male, MC: male castrated, FS: female spayed, MPV: molnupiravir, GS: GS-441524
